# Real sequence effects on the search dynamics of transcription factors on DNA

**DOI:** 10.1038/srep10072

**Published:** 2015-07-08

**Authors:** Maximilian Bauer, Emil S. Rasmussen, Michael A. Lomholt, Ralf Metzler

**Affiliations:** 1 Institute for Physics & Astronomy, University of Potsdam, 14476 Potsdam-Golm, Germany; 2 Department of Physics, Technical University of Munich, 85747 Garching Germany; 3 MEMPHYS—Centre for Biomembrane Physics, Department of Physics, Chemistry, and Pharmacy, University of Southern Denmark, Odense, Denmark; 4 Department of Physics, Tampere University of Technology, 33101 Tampere, Finland

## Abstract

Recent experiments show that transcription factors (TFs) indeed use the facilitated diffusion mechanism to locate their target sequences on DNA in *living* bacteria cells: TFs alternate between sliding motion along DNA and relocation events through the cytoplasm. From simulations and theoretical analysis we study the TF-sliding motion for a large section of the DNA-sequence of a common E. coli strain, based on the two-state TF-model with a fast-sliding search state and a recognition state enabling target detection. For the probability to detect the target before dissociating from DNA the TF-search times self-consistently depend heavily on whether or not an auxiliary operator (an accessible sequence similar to the main operator) is present in the genome section. Importantly, within our model the extent to which the interconversion rates between search and recognition states depend on the underlying nucleotide sequence is varied. A moderate dependence maximises the capability to distinguish between the main operator and similar sequences. Moreover, these auxiliary operators serve as starting points for DNA looping with the main operator, yielding a spectrum of target detection times spanning several orders of magnitude. Auxiliary operators are shown to act as funnels facilitating target detection by TFs.

Ever since the publication of the Luria-Delbrück model on bacterial resistance due to pre-existing mutants^1^ computational approaches to the dynamics of biological cells have contributed significantly to the advance of quantitative intracellular and cell population dynamics. Apart from the Luria-Delbrück model and its modifications^2^, the facilitated diffusion model has become a key to the understanding of genetic regulation in prokaryotes. Following the observation of Riggs and co-workers^3^ that *in vitro* lac repressors—one specific regulatory DNA binding protein commonly called transcription factors (TFs)—find their specific target sequence (operator) on E. coli DNA at a surprisingly high rate, scientists have examined the properties of the search of TFs for their target sequence. Early studies of Richter and Eigen^4^ were extended in the seminal work by Berg, Winter and von Hippel^5^. Their facilitated diffusion model explained the high association rates of TFs as a result of repeated rounds of diffusion in the bulk solution and intermittent sliding along the DNA. Interest in this model rekindled a decade ago^6^,^7^,^8^,^9^,^10^,^11^ along with novel single molecule experiments confirming the facilitated diffusion model *in vitro*^12^,^13^ and in living cells^14^,^15^,^16^.

Recent refinements of the facilitated diffusion model address molecular crowding effects both in the cytoplasm—reducing the TF-diffusivity—and along the DNA, where other (non-specifically) bound proteins impede the sliding motion of the TFs^17^,^18^,^19^,^20^,^21^. To account for the speed stability paradox^22^ TFs are believed to switch between the search state, in which the TF shuttles quickly along the DNA but is insensitive to the target, and the low-diffusivity recognition state, in which the particle is able to detect its target sequence^23^,^24^,^25^,^26^,^27^,^28^. The active role of spatial DNA conformations was unveiled both experimentally and theoretically^29^,^30^,^31^,^32^,^33^. Finally, the fact that genes, that interact via local TFs, are statistically proximate along the prokaryotic genome (colocalisation) was argued to be due to the increased interaction rates (rapid search hypothesis)^34^,^35^,^36^. In line with the increasing knowledge of the microscopic details of gene regulation many computational studies appeared that go beyond the typical idealisations^19^,^37^,^38^.

Motivated by recent experiments showing that on encounter the target operator is not detected with certainty by a TF sliding along the DNA^15^, we here combine theoretical and simulations analyses to quantify the sliding motion of a TF along the real nucleotide sequence of a common E. coli strain in the presence of crowding proteins on the DNA. We establish a model including search and recognition states of the TF in combination with the barrier discrimination model^10^,^24^ with a position weight matrix (PWM) based binding energy approach^39^. We also include looping effects—as often studied in thermodynamic models^40^—in the present model: the TF, for instance, the lac repressor dimer, can simultaneously bind to two operators, mimicking the intersegmental transfer mechanism^5^,^9^,^10^.

## Blockers and movers, and the role of auxiliary operators

**Figure 1:**
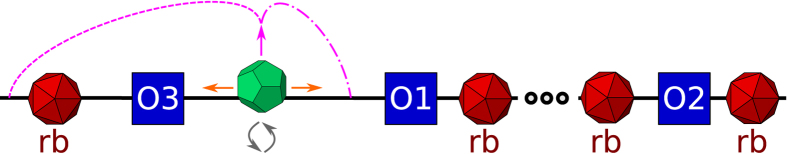
Scheme of TF search process along DNA (black line), which is partitioned by non-specifically bound roadblocks (red symbols). When TF (green symbol) is bound to DNA in the search mode, it can slide to a neighbouring position (orange arrows to the left and right) or interconversion between search and recognition state occurs (grey arrows below TF). Finally, dissociation (pink arrow) may lead to re-association nearby (dash-dotted line) or onto another segment (dashed line). The main and auxiliary operators (targets for TF binding) are shown as blue rectangles.

We describe the sliding motion of a TF for its target operator along DNA, on which *N*_block_ other proteins are bound, so-called blockers or roadblocks^18^. We focus on immobile blockers, keeping in mind that mobile blockers may add another layer of complexity^41^. The *N*_block_ non-overlapping blockers are positioned randomly and partition the DNA into *N*_block_+1 intervals. We assume that the TF cannot by-pass the blockers, see Fig. 1. Where the DNA is not occupied by a blocker, the TF can bind to the DNA in two orientations. In the case of palindromic sequences the binding energies in both orientations are equal (see also the score values in Methods).

We first focus on the processes in the target region carrying possible binding positions between the two nearest roadblocks to the left and to the right of the main operator 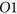. Such roadblocks could be proteins like H-NS or HU^42^. We only consider configurations in which the main operator is accessible. From both simulations and an approximate analytical approach we determine the probability 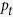 that the TF detects the target in the correct orientation before dissociation. The TF starts from a random position in this target region.

## Simulation scheme

We focus on base pairs 359,990 to 370,010 of E. coli strain K-12 MG1655 from ecocyc.org^43^, comprising the genes lacA, lacY, and lacZ as well as the three operators 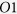, 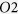, and 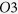, to which the lac repressor (LacI) can bind^44^. The sequence length is 10,021 base pairs (bps). Since the binding motif of LacI covers 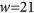 bps we obtain 10,001 possible binding positions in two orientations. We choose 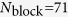 blockers of size 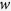 to match the occupation fraction of Tabaka *et al.*^21^.

The general simulation scheme is depicted in Fig. 1. At each position the TF can be either in the loosely bound search state or in the tightly bound recognition state. In the search state the TF has four possible actions: the particle can move to the left or to the right, it can dissociate, or it can change to the recognition mode at its position. If the latter occurs at the position of the main operator 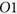, the corresponding time is saved as a first target detection. We later deal with dissociation from the DNA. Once in the recognition mode, we assume that the binding is so tight that the TF cannot move to neighbouring positions. As looping is neglected in this first, linear version of the model, its only option is to return to the search state at this position. The rates at which these transitions occur depend on the energetic barriers that need to be crossed during the internal protein dynamics. These are determined by the standard Gillespie algorithm^6^,^45^. Methods contains a detailed description of the simulations. Times are measured in units of the inverse attempt rate 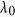 from Eq. (6) in Methods.

**Figure 2:**
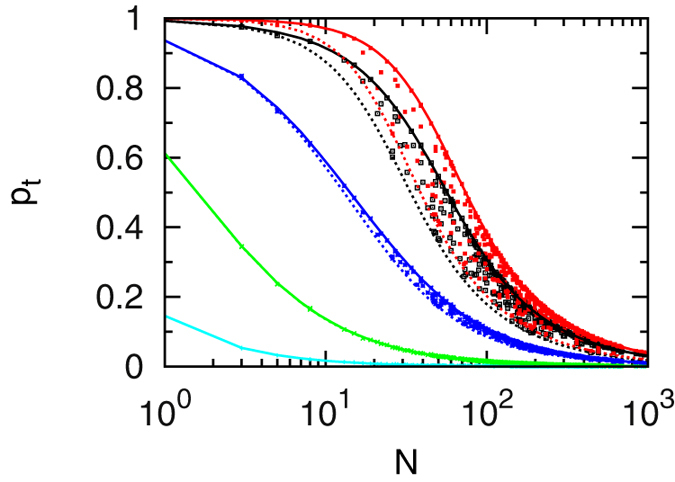
Probability 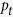 to detect the target before dissociation as function of the target region length 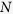. Symbols: simulations using 500 different configurations with 50,000 runs for each. Lines: simplified theoretical model with a centred target (full lines) and a target at the boundary (dashed lines). Parameters (in units of 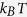): 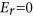, 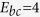, 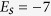, 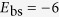. Colours: cyan (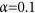), green (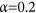), blue (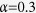), black (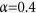) and red (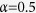).

The energy 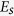 in the TF search state and the barrier 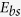 for sliding to a neighbouring base pair are assumed to be independent of the DNA sequence^10^,^46^. The barrier 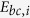 to switch to the recognition state and the associated TF energy 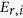 depend on the binding score (Methods) of the underlying sequence at the TF position 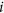. We express 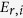 and 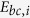 with respect to the reference scores 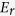 and 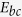, and we assume a linear relationship with the score at the specific position 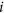,
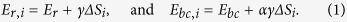
Here 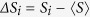 is the difference between the score at position 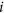 and the average score in the data set. 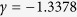 is a proportionality factor (Methods). The volatility parameter 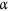 tunes the sensitivity of 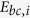 to the DNA sequence. If 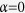 the barrier height does not change with the sequence and therefore this corresponds to blind testing of the sequence. If 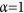, an induced fit mechanism is at work. The closer the probed sequence is to that of the target, the faster the TF switches to the target-sensitive recognition mode since the barrier height changes exactly as much as the energy in the recognition mode. To obtain the target detection probability before dissociation shown in Fig. 2 (see Results), 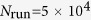 independent simulations starting from random positions in the target region were performed and it was counted in how many cases the target was reached. As we show here our model (1) for the energy score relation together with the additional element of the volatility 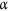 elucidate the role of the sequence sensitivity in the speed stability tradeoff of TF search processes.

## Theoretical approach

We compare the simulations results of Fig. 2 to a theoretical model based on a target region with 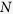 possible binding positions. For mathematical details see Methods.

The fundamental parameters are the sliding rate 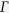 to neighbouring positions, the rate 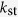 of a conformational switch to the recognition mode at the target site resulting in direct target detection, and the dissociation rate 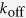 from any site. At all non-target positions we assume constant rates for the changes between recognition and search modes, denoted by 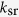 and 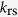. We place the target at bp 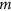 and the TF starts at a random position. As detailed in Methods these quantities determine the mean target detection time 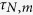 (see below) and the probability to reach the target before dissociation 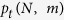, written as
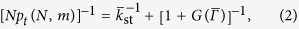
where 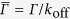 and 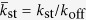. The function 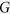 is defined via a series expansion in Eq. (17) (Methods). For 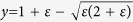 with 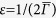, we find that
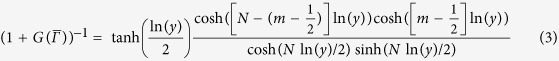
obtained by Kolomeisky *et al.*^47^,^48^ and studied experimentally in Ref. [49]. Thus Eq. (2) extends the result of Refs. 47,48 to the more general case when the target is not detected with 100% efficiency, as revealed in recent experiments^15^. Introducing the ratio 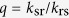, the mean search time 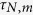 is (see Eqs. (9, 10, 11, 12, 13, 14, 15, 16, 17) in Methods)
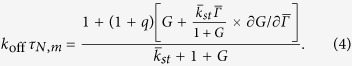


## Results I

### Target detection probability

Simulations results for the target detection probability 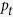 are shown in Fig. 2 for five 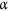 values between 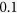 and 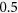. We do not consider larger 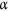 values since already for 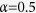 there is no longer an energy barrier to be crossed at the target site and thus no more changes are observed. Lines of matching colour in Fig. 2 are results of the analytical model, Eq. (2). The target is either centred (full lines) or located at the boundary of the target region (dashed).

The simulated data scatter nicely between the two limiting theoretical lines for centred and boundary target positions over three orders of magnitude in the size 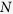 of the target region. 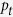 decreases monotonically with 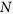, as large target regions on average imply longer paths which have to be traversed en route to the target, implying a higher risk to dissociate. Larger 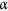 values, corresponding to a searcher which checks more often for the target, lead to a higher detection probability. Another effect of 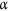 concerns the influence of the target position. For small values of 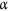 the corresponding curves nearly coincide, i.e., there is no significant target position dependence. For higher 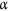 values, centred targets effect a substantially higher detection probability as the full lines lie above the corresponding dashed ones. Thus, only when the target detection probability on an individual encounter reaches substantial values, a suitable position of the target pays off.

We see that for the target detection probability the theoretical model, in which all energies on non-target sites are replaced by average values, nicely reproduces the results of the simulations based on sequence specific binding energy values.

### Target detection time

**Figure 3:**
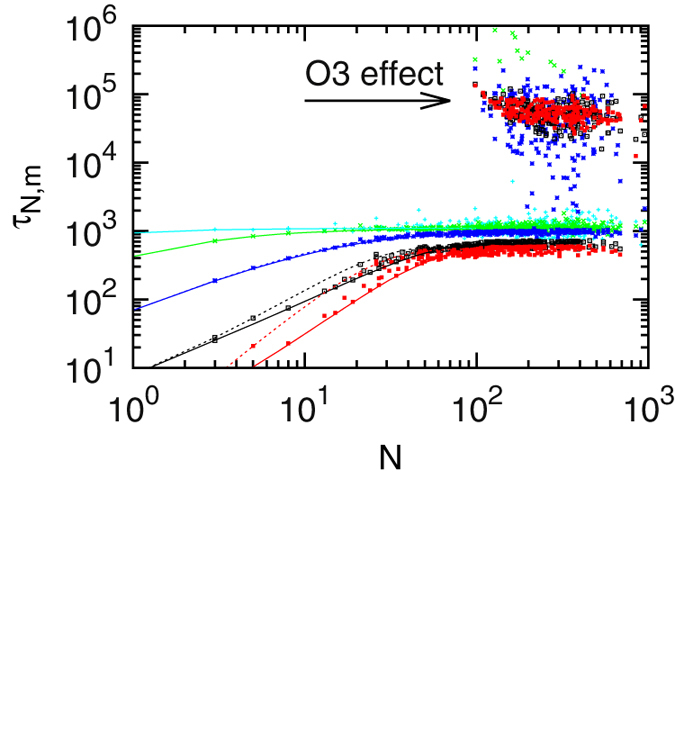
Mean first target detection time at 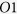 as function of the target region size 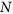. Symbols: simulations. Lines: theoretical model with a centred target (full lines) and with a target at the boundary (dashed lines). For small 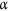 values dashed and full lines nearly coincide. Colours as in Fig. 2. Due to the presence of the auxiliary operator 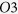 for *N* ≳ 100 a second branch of results emerges. Parameter values (in 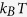): 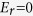, 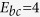, 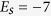, 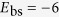.

In Fig. 3 the mean detection times 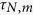 to the target are shown for the same 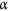 values used in Fig. 2. Since the particles can dissociate, 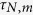 is a conditional time: given that the particle detects the target with the probability shown in Fig. 2, at what time will this occur on average. The symbols in Fig. 3 show the simulations results, the lines correspond to the theoretical model with a centred target (full lines) and a target at the boundary (dashed).

The features of Fig. 3 fall into two cases. For *N* ≲ 100, as with the detection probabilities above the simulations agree well with the theoretical model for all 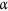 values. Again, a clear ordering with 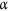 occurs: volatile TFs (large 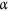) find the target quicker than nearly blind TFs with 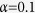 (cyan). Moreover, only in the case of large 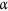, when individual encounters with the target have a substantial probability for target detection, the target position comes into play (e.g., for the red lines). This is one of our central results.

**Figure 4:**
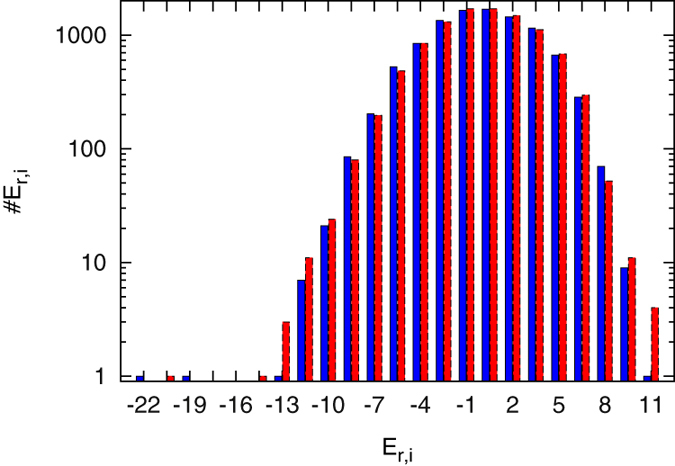
Logarithmic histogram of energy values in the recognition mode at all 10,001 positions in both orientations (blue and red) for 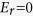, 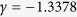.

For 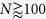, apart from simulations data consistent with the theoretical lines a second branch of results appears with target detection times nearly two orders of magnitude longer than expected. This effect can be rationalised by the presence of the auxiliary operator 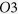 in the target region. It resides 92 nucleotides away from the main operator 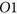 such that only target regions with a size larger than that can contain both operators^1^. If both operators are in the target region, the TF can change to the recognition mode at the auxiliary operator and thus become trapped away from the main operator. Such time consuming checks for the target may occur at any non-target position. However, at 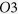 this is particularly severe since it has a rather strong binding energy (see Fig. 4). The gapped energy spectrum yields search times which are way above the values of the theoretical model, since the latter assumes all non-main target sites to be energetically equivalent.

Inspection of the upper branch of the results in Fig. 3 indicates that it barely contains data obtained with small 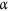 values (cyan and green). This can be explained by comparison with Fig. 2: in these cases even the probability to detect 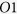 is rather small. This effect is even more pronounced for the considerably weaker 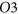. However, when such TFs change to the recognition state at the auxiliary operator, they will spend more time there than particles with a larger 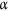, since these face a larger barrier to be crossed (Eq. (1)). As not all target regions of size *N* ≳ 100 contain the auxiliary operator, the lower branch of results still coexists. Here the conditional target detection time increases with 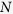 but levels off to a plateau.

Conversely, for rather volatile searchers (red data points) in regions comprising both operators, for 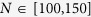 there is a slight tendency that the mean search time decreases with 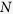. This results from the fact that these regions, which are only marginally longer than the distance between the two operators, by definition have both operators near the boundaries. This yields longer search times, similar to the case of shorter target regions, for which the dashed lines are always above the corresponding full lines in Eq. (3). We consider the influence of the location of the operators with respect to the non-specific blockers in more detail in the following paragraph.

### Preference of O1 over O3

**Figure 5:**
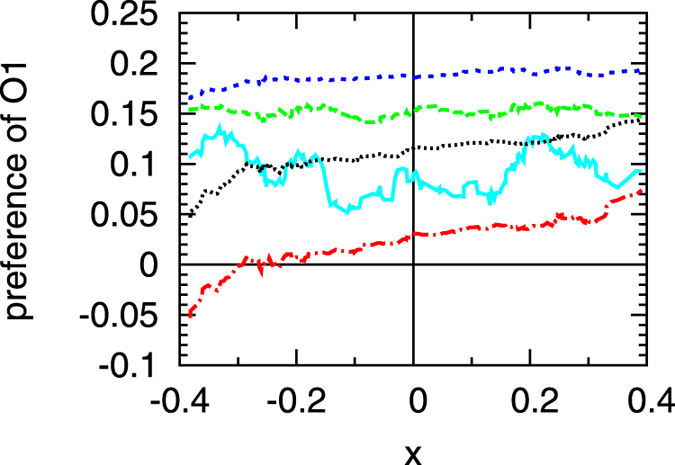
Preference of first detecting 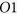 and not 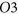 as function of the centrality 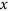 of the position of the two operators. Data constitute a moving average over each neighbouring 21 data points. Colours as in Figs. 2 and 3: 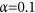 (full cyan line), 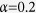 (green, long dashes), 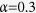 (blue, short dashes), 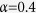 (dotted, black) and 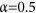 (red, dot-dashed).

In the hypothetical situation of two equally strong operators in the target region, only their relative position in the target region would influence which one of them is more likely to be detected first. The biologically relevant situation considered here with two different operators is more subtle. When both 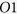 and 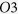 are in the target region we registered which one was detected first. The preference for 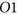 shown in Fig. 5 is given by the probability that 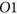 is detected first. The shift by 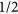 leads to positive values when the probability is larger to detect 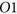 first.

To single out geometrical effects, the axis 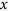 quantifies which of the two operators is more central in the target region and thus has—from a geometric point of view—higher chances to be hit first. We define 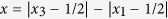, where 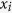 denotes the relative position of operator 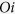, 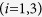 in the target region. The 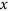 values range between 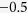 and 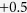, positive values corresponding to a favourable position of 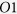.

As expected, since 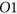 is the stronger operator, most of the data points are positive. For small 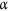 values (cyan and green in Fig. 5) it is more probable to detect 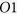 first, but the relative positions of the two operators are not significant. Increasing the volatility from 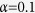 to 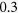 leads to a monotonic increase in the accuracy of discrimination between the two operators. For even larger values of 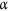 this accuracy decreases, since now the particle checks for the target often enough to detect the auxiliary operator with sufficient probability. Then, geometric effects become more important, as seen from the increasing slope of the black dotted line for 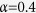 and the red dot-dashed line for 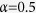. In the latter case, some negative values of the preference are observed, indicating that a volatile TF is more likely to detect the auxiliary operator first, if its position is much closer to the centre of the target region.

Intermediate 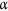 values enable the TF to detect the main operator first, without losing time from binding to the auxiliary operator. In terms of the search model presented so far, the occurrence of 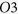 appears like a design bug instead of a useful feature, since it delays the detection of the main operator. We now show that auxiliary operators in a more realistic scenario indeed act as funnels for TFs towards the main binding site.

### Auxiliary operators make sense in presence of looping

As evident from Figs. 3 and 5 the presence of the auxiliary operator 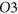 in the target region significantly influences the rate of target detection. In an extension of our model several configurations can be distinguished depending on whether or not the two auxiliary operators are accessible. In a living cell the occupation with non-specific binders and thus the probability for a particular blocker conformation change in time.

To model the complete search process of a TF with two binding motifs such as LacI, we consider what happens after a dissociation from DNA. After dissociation the time spent in 3D is assumed to be exponentially distributed with mean time 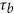. For the jump length 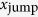
—like all the following lengths measured in bps—on the DNA effected by 3D excursions we assume the cumulative distribution
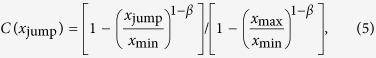
characterised by the minimal jump length 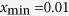, the maximal jump length 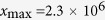
—corresponding to half the E. coli genome size, and the scaling exponent 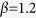 characterising the looping properties of the repressor. Scaling laws of the form 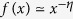 for the length 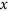 stored in a random loop formed by a polymer chain occur due to the equivalence of polymers to random walks. For a random chain in three dimensions 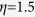, while in the presence of excluded volume interactions the exponent increases to 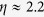
50,51,52. Here we chose the lower exponent 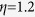 following the data by Priest *et al.*^53^. To obtain the cumulative distribution (5), we integrate the power law 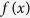 in between the lower and upper cutoffs 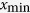 and 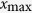, and normalise this expression. Note that our results are not overly sensitive to the exact value of the exponent 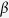, as in the free energy it corresponds to a logarithmic dependence on 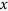.

Here we assume that a power law similar to Eq. (5) also applies to the jump statistics. Whenever the particle jumps out of the 10 kbps range that we study, we place it at a random position in our system, mimicking the complete loss of correlation with the dissociation position for long jumps. Unlike during sliding motion, it can change the orientation during a 3D relocation. To simplify matters we coarse-grain events outside the target region, since we are not interested in the sliding motion far away from the target. We then first simulate the mean dissociation times from all 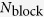 regions that do not contain the target. To this end, simulations are performed as outlined in the paragraph *Simulation scheme*, where the code is run 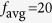 times multiplied by the length of the corresponding interval measured in bps to guarantee reasonable statistics.

Whenever the TF detects and binds to one of the auxiliary operators, apart from returning to the search state at this position there is the possibility to form a DNA loop with 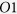. For this event to occur, an initiation time is drawn from an exponential distribution with mean 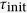, which is assumed to be the time needed to form a non-specific complex with the target region. To keep the number of parameters as low as possible we assume these initiation times to be equal for both auxiliary operators. To the loop initiation time we add a time lag, since after landing with its second half in the target region, the TF has to actually detect the main operator. The latter is obtained from a simulation as defined in *Simulation scheme*. The same process is possible the other way round: starting from binding to the main operator and, before switching to the search mode, closing a loop with one of the auxiliary operators. To simplify matters we do not model direct looping between the auxiliary operators. The times for releasing a loop are calculated similarly to the mean dissociation times above. We now study the full model with looping for 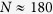.

## Results II

### Influence of the volatility

**Figure 6:**
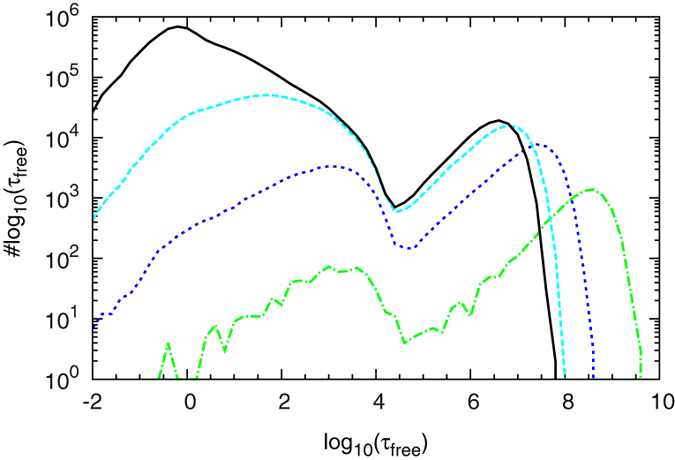
Distribution of time spans 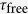 during which the operator is free of repressor in a system without looping. The abscissa shows the logarithms of the time spans during which the operator is accessible such that bins at larger 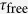 values are wider. Parameters: 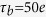, 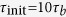. Here 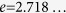 is Euler’s number, further information on the used parameters is provided in the Methods. The total simulation time is 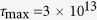. We use four values 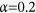 (green, dash-dotted), 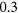 (blue, short dashes), 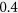 (cyan long dashes) and 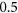 (black, full line).

Of particular biological interest are the time spans 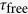 during which the operator is unoccupied, as in these intervals RNA polymerase can bind to the promoter and start transcription. We start with a conformation in which looping is precluded by blocking both auxiliary operators with non-specific binders. In Fig. 6 the distribution of 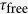 is shown for four values of the volatility parameter 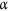.

In all cases we obtain two distinct peaks separating a short and a long time scale. For increasing values of 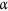 the first peak, located at around 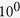 time units, grows relative to the second one, located at around 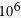 time units. Since the total simulation time was fixed, the total number of events grows as well: The peak at short times is due to events when a TF, after switching from the recognition to the search mode, performs just a few sliding steps before returning to the recognition state at the target. Conversely, the long time peak corresponds to events when a TF dissociates, possibly multiple times, from DNA and loses correlation with the unbinding position, and thus leads to long time spans, in which the target operator is vacant. That the first peak gains in importance for larger values of 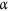 is due to the fact that, as seen above, the individual target detection probability is higher in that case.

We note that to initiate transcription, RNA polymerase must bind the promoter while the TF is not at the operator. If the repressor rebinds to the operator before an RNA polymerase manages to find the promoter, the cell does not “feel” these quick occupancy fluctuations and experiences only a single effective binding event of the repressor, and no transcription takes place (compare Ref. [54]).

### Influence of looping and the average time spent in 3D

**Figure 7:**
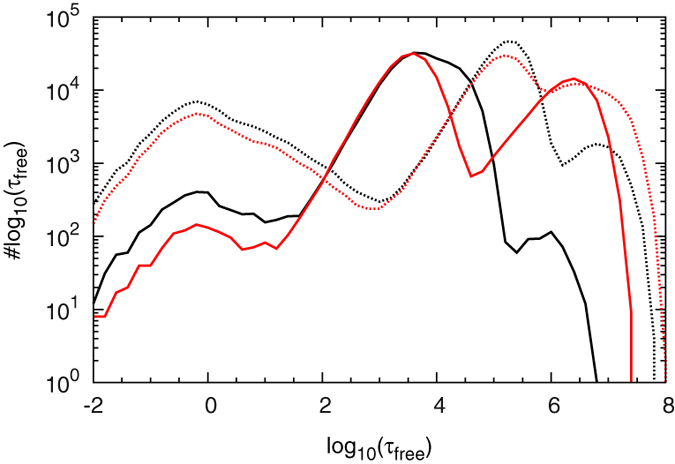
Distribution of 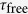 in systems where looping is possible involving 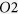 (black lines) and both 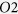 and 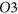 (red lines). Full lines: 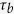, 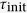, 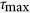 as in Fig. 6. Dashed lines: 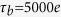, 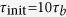. In all curves: 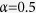.

We now choose a configuration in which the auxiliary operator 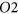 is vacant and we fix 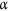 to a value of 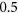. The corresponding results are shown by black lines in Fig. 7. Full lines are for the same values of 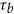 and 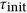 as in Fig. 6, dashed lines represent the case when both are ten times larger. We observe that both full lines still feature two peaks centred at 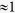 and 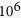 time units. Between these there appears a new peak at intermediate times. Given that the loop initiation time in this case is 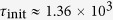, these events can be self-consistently interpreted as return events to the target due to looping: the DNA was looped between the main and an auxiliary operator, dissociates from the main operator and reestablishes the loop.

That the peak for fast rebinding events has a reduced size is due to the fact that our looping algorithm counts all fast fluctuations of the occupancy during which the loop still exists as a single long-lived event. In the simulations without looping these events appeared explicitly (Fig. 6). Accordingly, the remaining events in the reduced first peak correspond to target rebinding without an existing loop to an auxiliary operator. Given that fast rebinding has no biological meaning, looping introduces a new intermediate time scale, and typical return times to the operator are greatly reduced, resulting in improved repression. This agrees with the observations of Choi and co-workers according to which DNA looping enables the cell to regulate gene expression on many time scales via distinct forms of dissociation events^55^. Comparing this behaviour to the black dashed line in Fig. 7, when both 3D excursions and looping take around ten times longer, shows that both the looping peak and the rightmost peak are shifted to larger times underlining the physicality of our interpretation.

If both auxiliary operators are accessible and 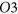 is in the target region (red lines in Fig. 7), the results are similar to the previous ones (Fig. 6). Three peaks are observed, and increase of 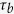 and 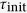 shifts the peaks—apart from the 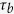-independent fast rebinding peak—to the right. There is one major difference between the two settings: When both auxiliary operators are accessible, the size of the third peak is nearly as large as the second one. Thus, very long return times occur more often when both auxiliary operators are present. The significant changes of the target search times in the presence of the auxiliary operators are our other central result.

## Discussion

One-dimensional sliding of a TF along the DNA is a vital ingredient of the facilitated diffusion model. Sliding is indispensable in the final step of the search for the specific binding site by the TF, namely, the recognition of the binding sequence. For a real bacterial DNA sequence we here analyse in detail the dynamics of the TF sliding in a region around the main operator in the presence of roadblocks, e.g., proteins like H-NS or HU^42^. For a minimal set of parameters we unveil the role of the density of the roadblocks and the DNA sequence on the detection speed of the target sequence. Our results underline the special role played by auxiliary TF operators. These auxiliary operators act as a funnel for the TF to facilitate the target search in the nucleotide sequence.

More specifically, combining a simplified theoretical model and simulations we follow a TF moving in a region around the main operator delimited by two non-specifically bound roadblocks while switching between a search mode, in which it shuttles along the DNA while being blind to the target, and a recognition mode, in which it cannot move along DNA but which is essential to detect the target. The interconversion rates depend on the underlying sequence. Motivated by recent experiments showing that not every target encounter leads to detection of the target sequence^15^, we interpolated between the extreme cases of nearly blind switching between the modes and an induced fit situation, in which the energetic barrier to be crossed changes as much as the specific binding energy. Numerical results for the probability to detect the target before dissociation and for the mean detection time demonstrate impressive agreement with our theoretical model (see Fig. 2 and upper branch in Fig. 3), as long as no further binding sites of similar strength are present. If an auxiliary operator is within the target region, an intermediate rate of checking for the target yielded the highest accuracy in discrimination between main and auxiliary operators. However, while auxiliary binding sites act as traps in the simplified model, in the more realistic situation when DNA looping is allowed, they can be seeding points for the formation of loops joining two operators. In the second part we therefore included looping in the simulation. For our parameters, this leads to quick rebinding events to the main operator and thus increases significantly the local effective TF density, in accordance with classical observations. This approach can be easily transferred to other TFs with known binding motif.

Given the fairly large number of the parameters involved and the complexity of the dynamics conveyed by the broad range of apparent time scales, definite quantitative statements of this problem are hard to give. Furthermore, the target search here was modelled for a single TF, while in a living bacteria cell approximately a dozen lac repressors perform this task simultaneously. Additionally, other TFs could partially block the specific binding site of the TF under consideration, and could impede the establishment of the lac specific loop. As recent studies showed (for instance, see Ref. [20]) the effects of additional binders are not always obvious and require careful analysis. Since the binding to the operator(s) is rather strong, it is questionable to assume that the TFs are independent and we face a multi-body problem. However, the concentration effects and the expression output in terms of the occupancy of all three operators were successfully studied in terms of thermodynamic models^40^ using similar language. As our simplified theoretical model for events in the target region yields such a good agreement with the numerical simulations and given that more and more quantitative experimental results appear, it seems to be a logical extension to equip thermodynamic models with rates obtained from our model presented herein. Moreover, the accessibility of the three operators could be modulated in time to mimic the mobility of nonspecific binders which can block the operators. In this spirit we believe that the results reported herein represent an important step forward toward the quantitative understanding of gene regulation in living prokaryotic cells, and form the basis for future, more detailed models.

## Methods

Here we describe the simulations method and the calculations for the above results.

### Numerical simulation of the simplified model

The TF is present in either the loosely bound search state or tightly bound recognition state. In the search state at position 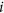 the TF can either slide to the neighbouring sites 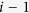 or 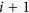 while remaining in the search state, it can dissociate or switch to the recognition state at the same position (Fig. 1). Such a switching event at the target site (the operator 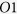) corresponds to detecting the target. This differs from the approach of Ref. [56], in which a further target detection step was used after changing to the recognition state at the target site. If the particle is next to a blocker and tries to move onto the excluded site, the move is cancelled. The standard Gillespie algorithm is used to draw the rates for the above events. A central role is played by the energetic barriers which need to be crossed, measured in units of 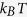 with respect to the unbound state of zero reference energy (similar to Refs. 22,57).

In the recognition state we assume the TF to be immobile. In the first version of the model without looping the TF can only return to the search state. Generally when going from state 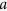 with energy 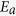 to state 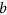 with energy 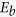, separated by an energetic barrier 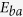, the rate, 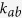 for this step is given by
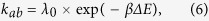
with 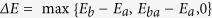. In absence of a barrier (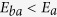) and when the energy of the final state is smaller than that of the initial state (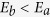) the reaction is assumed to occur with attempt rate 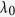, which is the inverse of the elementary time step in which all times are measured. To convert our results to real times, this time step can be related to the known 1D diffusion coefficient of a given TF. We note that our approach differs from the convention of Ref. [58,^59^], in which the specific binding barrier has to be crossed each time the TF slides to a neighbouring position.

We fix the energy difference between the specific binding energy at the main operator 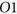 and the energy in the search state as 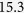 60. With the choice 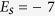 applied in the main text this implies 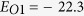 (Fig. 4). The proportionality factor 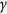 can be determined once all values of the score matrix are known via the above mentioned demand 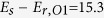 60.

### Score matrix

The score matrix is obtained from standard methods and calculated for both orientations in which the TF can bind: the PWM score 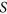 of a putative in the most general form is written as^61^
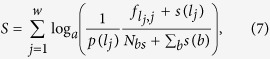
where 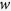 denotes the length of the binding motif, 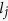 the nucleotide at position 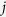 in the input sequence, 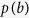 the background frequency of base 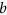, 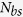 the number of known binding sites, and 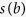 a pseudo-count function.

In the following we stick to the convention used by Vilar^62^, namely, 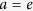 (where 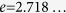 is Euler’s number), 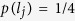 for all 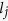 (all nucleotides appear with equal probability) and 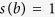 for all 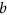 (we use the same pseudo-count function for all four types of nucleotides). Given that there are 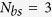 known operators to which the repressor binds (commonly denoted by O1, O2 and O3), this yields
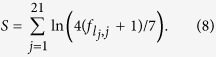


For the three known operator sites the scores are^62^: 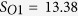, 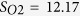, and 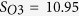. A histogram of the energy values in the recognition state for the 10,001 binding positions surrounding the 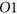 operator is shown in Fig. 4, where 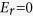 and 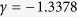 (a proportionality constant translating score differences into energetic differences) were chosen such that 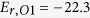. At the lower end of the energy spectrum the three operators can be recognised. Note that there is an energetic gap to all other binding sites, see the discussion of such a gapped situation in Ref. [63].

### Simplified theoretical model

The simplified theoretical model includes 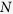 possible binding positions, 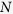 being an odd number. This way a central node exists, but an analogous calculation can be done for even 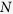. Applying the scheme of possible reactions we have the following differential equations for the probability density 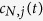 of TFs in the search state at base pair 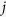 at time 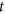 and the corresponding probability density 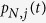 of TFs in the recognition state, when the TF is at bp 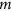,
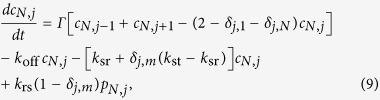
and
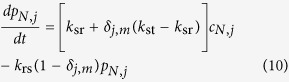


This set of equations is more conveniently treated in Laplace space with respect to time, where we denote the variable conjugate to 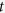 by 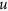 and the corresponding functions with a tilde. For convenience we omit the explicit argument 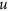 in the following.

If the particle starts its motion in the search state, initially the probabilities 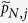 vanish. This is due to the simple proportionality between 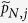 and 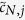,
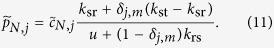


In particular, at the target site 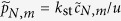, and at all other sites 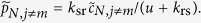 Solving this system of equations amounts to finding the solution of a tridiagonal matrix system. Of particular interest is the probability at the target site encoding the Laplace transform of the flux to the target,
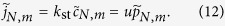


In the following we introduce a temporary additional index for 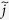, 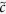 and 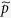 denoting the node on which the particle starts, taken to be 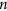. With the auxiliary function 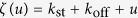 the flux to the target becomes
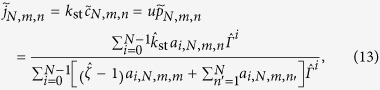
where quantities with a hat are obtained by dividing the corresponding quantities without hat by the auxiliary function 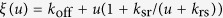. The parameters 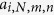 are given by
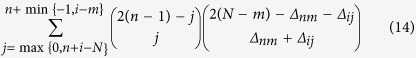
for 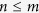, and
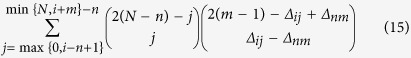
for 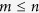. 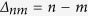 and similarly 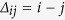.

For a homogeneous initial distribution we omit the last index for the starting position of the TF and
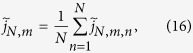
which can be Taylor expanded of up to first order in 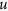 yielding the probability 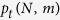 to reach the target before dissociation as well as the mean (conditional) target detection time 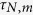 given by Eqs. (2) and (4). The function 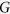 is defined by the series expansion
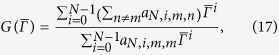
where 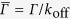. Note that the auxiliary function 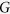 does not depend on the target detection rate 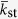, but only on the geometry of the system via 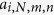 and on the hopping dynamics encoded in 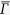. For a centred target, 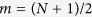, and in the limit 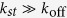 the target detection probability simplifies to
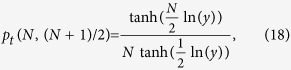
reminiscent of Ref. [64].

For the conditional mean search time for a centred target in the limit of vanishing dissociation rate 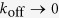, we obtain 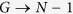 and 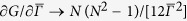, such that via Eq. (4),
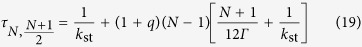


In this limit the existence of the recognition state away from the target simply slows down the mean target detection time via the prefactor 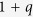 of the second term. In the limiting case 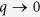, when the recognition state is never entered unless the particle is on the target site, this result reduces to the classical solutions for incoherent exciton hopping, 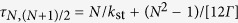 65.

## Additional Information

**How to cite this article**: Bauer, M. *et al.* Real sequence effects on the search dynamics of transcription factors on DNA. *Sci. Rep.* **5**, 10072; doi: 10.1038/srep10072 (2015).
