# Corrigendum: Real sequence effects on the search dynamics of transcription factors on DNA

**DOI:** 10.1038/srep13045

**Published:** 2015-08-26

**Authors:** Maximilian Bauer, Emil S. Rasmussen, Michael A. Lomholt, Ralf Metzler

Scientific Reports **5**: Article number: 1007210.1038/srep10072; published online: 07082015; updated: 08262015

The Acknowledgements section in this Article is incomplete.

“RM acknowledges the Academy of Finland for support within the FiDiPro scheme.”

should read:

“RM acknowledges the Academy of Finland for support within the FiDiPro scheme. We acknowledge financial support of the Deutsche Forschungsgemeinschaft (DFG) and the Open Access Publication Fund of the University of Potsdam.”

